# Underwater Image Enhancement Using a Diffusion Model with Adversarial Learning

**DOI:** 10.3390/jimaging11070212

**Published:** 2025-06-27

**Authors:** Xueyan Ding, Xiyu Chen, Yixin Sui, Yafei Wang, Jianxin Zhang

**Affiliations:** 1 School of Computer Science and Engineering, Dalian Minzu University, Dalian 116600, China; dingxueyan@dlnu.edu.cn (X.D.); xiyuchen1120@163.com (X.C.); syx5208042@163.com (Y.S.); 2 Research Center of Multimodal Information Perception and Intelligent Processing, Dalian Minzu University, Dalian 116600, China; 3 School of Information Science and Technology, Dalian Maritime University, Dalian 116026, China; wangyafei@dlmu.edu.cn

**Keywords:** underwater image enhancement, diffusion model, adversarial learning, attention mechanism

## Abstract

Due to the distinctive attributes of underwater environments, underwater images frequently encounter challenges such as low contrast, color distortion, and noise. Current underwater image enhancement techniques often suffer from limited generalization, preventing them from effectively adapting to a variety of underwater images taken in different underwater environments. To address these issues, we introduce a diffusion model-based underwater image enhancement method using an adversarial learning strategy, referred to as adversarial learning diffusion underwater image enhancement (ALDiff-UIE). The generator systematically eliminates noise through a diffusion model, progressively aligning the distribution of the degraded underwater image with that of a clear underwater image, while the discriminator helps the generator produce clear, high-quality underwater images by identifying discrepancies and pushing the generator to refine its outputs. Moreover, we propose a multi-scale dynamic-windowed attention mechanism to effectively fuse global and local features, optimizing the process of capturing and integrating information. Qualitative and quantitative experiments on four benchmark datasets—UIEB, U45, SUIM, and LSUI—demonstrate that ALDiff-UIE increases the average PCQI by approximately 12.8% and UIQM by about 15.6%. The results indicate that our method outperforms several mainstream approaches in terms of both visual quality and quantitative metrics, showcasing its effectiveness in enhancing underwater images.

## 1. Introduction

During human exploration of the oceans, the quality of underwater images directly affects the accuracy with which both humans and machines compute, recognize, analyze, and interpret visual data. However, due to the different absorption rates of light waves and the existence of refraction and scattering problems, underwater images generally have color distortion, blurred details, and other problems [1]. Underwater image enhancement technology is key to solving this problem, which is of great significance to the fields of underwater archaeology, marine biology research, and marine resource development [2,3]. Compared to other image enhancement tasks, the complexity and diversity of underwater environments present a unique set of challenges for underwater image enhancement. Specifically, the attenuation of light in water is closely related to its wavelength; the longer the wavelength, the faster the attenuation. Thus, red light, which has the longest wavelength, attenuates the fastest, while blue and green light attenuate more slowly, causing the color distortion commonly observed in underwater images. Moreover, suspended particles in water can cause both forward and backward scattering, resulting in blurriness, reduced contrast, and increased noise in underwater images. Image enhancement technologies are effective in mitigating light attenuation and color distortion in underwater environments, thereby improving the image quality of underwater robots or detection equipment and enhancing the accuracy of image analysis.

In recent years, significant transformations in underwater image enhancement have been driven by remarkable advancements in computer vision technology. Deep learning methods, with their data-driven strategies, autonomously learn complex image features and relationships, making them highly effective for underwater image enhancement. Generative adversarial networks (GANs) [4] produce notably clearer and more realistic images, making them the preferred model for underwater image enhancement tasks in recent years. GAN leverages its adversarial mechanism to learn the image distribution of the target domain, making it well-suited for image enhancement tasks in various underwater environments. However, GAN tends to optimize overall image quality, often overlooking small-scale features (such as fine textures and edges), resulting in less realistic local details [5]. Instead, diffusion models excel at modeling probability distributions, allowing them to progressively denoise and restore image details [6]. This approach shows significant advantages in reconstructing fine-grained details and preserving natural textures, but struggles with global consistency, such as overall color balance and lighting correction, which limits their effectiveness in underwater image enhancement. Underwater images often require enhancements to both local details and global features. Therefore, we propose a diffusion model-based underwater image enhancement method using an adversarial learning strategy. It fully leverages the strengths of GANs and diffusion models to enhance the quality and stability of generated images, addressing some of their individual limitations. The main contributions of this paper are summarized as follows:

- We introduce an adversarial diffusion model for underwater image enhancement, named ALDiff-UIE. It employs a diffusion model as the generator to produce high-quality images through its iterative denoising steps and utilizes a discriminator to evaluate and provide adversarial feedback to refine the generated images. The diffusion model guides the generation of local details, while feedback from the discriminator enhances global features, resulting in more globally consistent and visually realistic generated images.
- We developed a multi-scale dynamic-windowed attention mechanism, designed to effectively extract features at multiple scales and implement self-attention within windows along both vertical and horizontal dimensions. This mechanism incorporates an agent strategy that optimizes the process of capturing and integrating information while reducing computational complexity.
- The comprehensive experimental results demonstrate that the proposed method significantly enhances image details and contrast. Across various standard evaluation metrics, the proposed method shows substantial performance improvement, further validating its effectiveness in the field of underwater image enhancement.

This paper is organized as follows: Section 2 discusses related work on underwater image enhancement methods, Section 3 presents the ALDiff-UIE method, and Section 4 concludes with experiments and results demonstrating the effectiveness of the proposed method. Section 5 and Section 6 summarize the strengths and weaknesses of the proposed algorithm and provide an outlook for future research work.

## 2. Related Work

In recent years, the rapid development of underwater exploration has driven researchers to actively engage in the study of underwater image enhancement. The main goal of underwater image enhancement is to improve the visual quality of degraded underwater images, providing more accurate and reliable data for subsequent observations and analysis. Currently, underwater image enhancement methods can be categorized into traditional methods and data-driven methods [7].

### 2.1. Traditional Methods

Traditional methods primarily rely on physical models or image processing techniques, utilizing prior knowledge or assumptions to enhance images. These methods can be broadly categorized into physical model-based methods and non-model-based methods.

Physical model-based methods rely on underwater image formation models, requiring the inverse operation of these models to restore clear images. For instance, Berman et al. [8] considered multiple spectral profiles of different water types and estimated the attenuation ratios of the blue–red and blue–green color channels, reducing the underwater image enhancement problem to a single-image dehazing task. Muniraj et al. [9] proposed an underwater image enhancement method by combining a color constancy framework and dehazing, in which the transmission map is estimated by analyzing the intensity differences between each channel. Li et al. [10] proposed a method for underwater image enhancement, PWGAN (physics-based WaterGAN), which utilizes both the scattering coefficient of underwater images and the depth information from clear images to fine-tune the outputs of two generative adversarial networks, effectively reducing color distortion and image blurring while restoring the details of the underwater objects. Most physical model-based methods rely on estimating background light and depth in underwater images. However, these methods are effective only in specific scenes, as physical features, such as prior knowledge, cannot be accurately estimated in more complex underwater environments, limiting the generalizability of these approaches.

Non-physical model-based methods enhance underwater images by directly modifying pixels using techniques that do not take the underwater image formation model into account. For instance, Li et al. [11] introduced a contrast enhancement algorithm based on prior histogram distributions. Xu et al. [12] presented a weak-light image enhancement fusion framework that combines multi-scale fusion with dual-model global stretching. Some studies are based on the Retinex theory, such as Zhang et al.’s [13] development of a multi-scale Retinex algorithm that combines bilateral and trilateral filtering for more precise image processing. Zhuang et al. [14] developed an underwater image enhancement method based on Bayesian Retinex, incorporating multi-order gradient priors for reflectance and illumination. Non-physical modeling methods perform image enhancement by modifying the pixels of an image, which is simple and easy to implement and low-cost, but ignores the optical properties of underwater imaging, resulting in commonly over- or under-corrected images after enhancement.

### 2.2. Data-Driven-Based Methods

In recent years, data-driven deep learning methods have gained significant traction in the field of underwater image enhancement, owing to their powerful feature-learning capabilities. These methods have shown great promise in advancing the state-of-the-art in underwater image enhancement. Deep learning-based underwater image enhancement methods can be broadly divided into four main categories, as follows: CNN-based methods, GAN-based methods, Transformer-based methods, and diffusion model-based methods.

CNN-based methods: Wu et al. [15] designed a two-stage underwater image convolutional neural network based on structural decomposition to enhance the texture information of the image by decomposing the underwater image into a high-frequency image and a low-frequency image. Jiang et al. [16] adopted a divide-and-conquer strategy, comprising a strong prior phase that decomposes complex underwater degradation into sub-problems and a fine-grained phase that employs a multi-branch color enhancement module and a pixel attention module to improve color accuracy and detail perception. Li et al. [17] proposed UWCNN, which is a lightweight CNN network with jointly optimized multinomial loss based on a priori knowledge of the underwater scene. Khandouzi et al. [18] proposed a coarse-to-fine end-to-end underwater image enhancement method combining classical image processing with lightweight deep networks, which achieves effective enhancement of underwater images through global-local dual-branching CNN, improved histogram equalization, and attention module.

GAN-based methods: These methods build an adversarial neural network comprising generators and discriminators, generating images through the competition between these components. Liu et al. [19] integrated features across different scales, enhancing the model’s capability to improve image quality. Islam et al. [20] employed an objective function aimed at capturing global content, style, color, and local texture comprehensively, facilitating a more thorough feature capture of the image. Zhang et al. [21] designed a layered, attention-intensive aggregation in the encoder and introduced inter-block serial connections to better adapt to complex underwater scenes. Chaurasia et al. [22] treated underwater image enhancement as an image-to-image translation task, and customized the objective functions to achieve effective conversion of color, content, and style. Guo et al. [23] proposed a Transformer-based generative adversarial network with a two-branch discriminator, generating more realistic colors while preserving image content.

Transformer-based methods: Transformer is a deep learning architecture based on self-attention mechanisms. Ren et al. [24] enhanced the U-Net architecture by embedding the Swin Transformer, improving the model’s ability to capture global dependencies and boosting its performance in underwater image enhancement tasks. Peng et al. [25] proposed a U-shaped Transformer and introduced a loss function that combines various color spaces. Khan et al. [26] proposed a multi-domain query cascaded Transformer network that integrates local transmission features and global illumination characteristics. Shen et al. [27] proposed a dual-attention Transformer that integrates channel self-attention and pixel self-attention mechanisms. This design significantly enhances underwater image processing by effectively capturing features and contextual information.

Diffusion model-based methods: Originally proposed by Sohl-Dickstein et al. [28] in 2015, the diffusion model has regained researchers’ attention in recent years with the introduction of the denoising diffusion probabilistic model (DDPM) [29] for image generation tasks. Unlike the encoding and generation processes of other generative models, DDPM breaks these processes into multiple steps, enabling precise approximation of small variations using a normal distribution. Lu et al. [30] proposed an improved network structure based on DDPM, introducing a dual U-net architecture for the image enhancement task, which effectively facilitates the transformation between two image data distributions. Tang et al. [31] introduced a conditional diffusion framework for underwater image enhancement, along with a lightweight Transformer-based neural network as a denoising network, which not only enhances image quality effectively but also reduces the runtime of the denoising process. Shi et al. [32] proposed a content-preserving diffusion model, utilizing the difference between original images and noisy images as input. Zhao et al. [33] proposed a physics-aware diffusion model that effectively utilizes physical information to guide the diffusion process.

Despite significant advancements in underwater image enhancement, a notable gap remains in effectively addressing both local and global image features simultaneously. Traditional methods, including both physical and non-physical approaches, face challenges in adapting to the complex underwater environment due to their reliance on prior knowledge and limited assumptions. While deep learning techniques, such as GANs and diffusion models, have shown promise, GANs prioritize global image quality at the cost of fine details, whereas diffusion models excel at recovering local features but struggle to maintain global consistency. Therefore, there is a clear need for a method that combines the strengths of GANs and diffusion models while incorporating advanced attention mechanisms to achieve more precise enhancement.

## 3. Proposed Method

**Figure 1:**
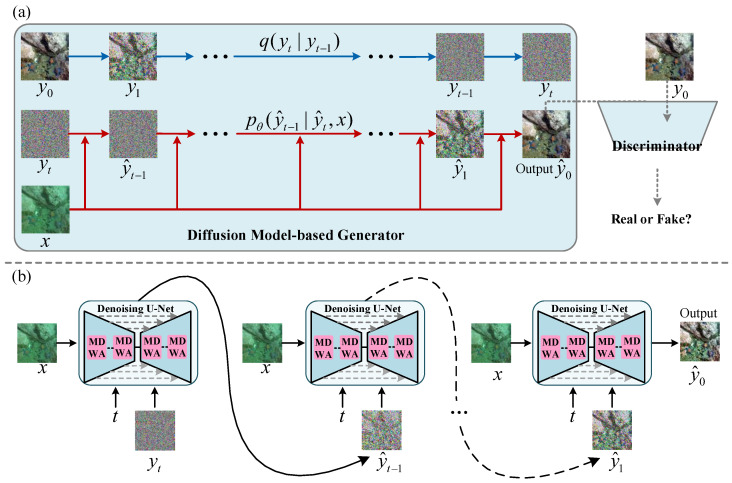
The diagram of the proposed method. (**a**) Provides an overview of the ALDiff-UIE framework, which consists of a diffusion model-based generator and a discriminator trained in a competitive framework. The generator utilizes a diffusion model that incrementally adds noise to the image in the forward process and removes it step-by-step in the reverse process to generate a new image. (**b**) Depicts the inference process of the proposed method, detailing how the framework produces enhanced underwater images. MDWA refers to the multi-scale dynamic-windowed attention module.

To obtain high-quality underwater images, we propose an underwater image enhancement method, named ALDiff-UIE. As shown in Figure 1a, the proposed method comprises two core components, that is, a diffusion model-based generator and a PatchGAN-based discriminator, trained in a competitive framework. The generator attempts to produce data, while the discriminator attempts to distinguish between real and generated data. The diffusion model is based on probabilistic modeling, where noise is incrementally added to the data during the forward process and then removed step-by-step in the reverse process to generate new data.

The generator employs the denoising process of the diffusion model to eliminate noise and enhance image details, with its inference process illustrated in Figure 1b. However, the randomness inherent in the generation process of diffusion models poses significant challenges for underwater image enhancement tasks. Therefore, during the denoising process, we use degraded underwater images to guide the model in generating enhanced images with similar high-level semantic information as the degraded images, achieving finer control. In contrast to traditional generators, our generator begins with Gaussian noise and gradually introduces structure and patterns until the generated data aligns with the desired distribution. This gradual process helps prevent issues related to pattern collapse.

### 3.1. Diffusion Model-Based Generator

The generator employs a diffusion model that incrementally adds noise to the data in the forward process and removes it step-by-step in the reverse process to generate new data. The forward process is a Markov chain in which Gaussian noises are gradually added to the input data over several time steps, driving the distribution of the data to converge towards a standard Gaussian distribution. This step is predefined and does not require learning. In contrast, the denoising process trains a neural network in a learnable manner, using pure noise as input and the degraded underwater image as a condition, enabling the model to progressively recover the distribution of the clear underwater image.

The core strategy of the generator is to learn a specific inverse process, denoted as $p_{\theta }\left(y_{0:T}|x\right)$, without altering the forward diffusion process $q(y_{1:T}|y_{0})$. The forward Markovian diffusion process *q*, which involves the stepwise addition of Gaussian noise, can be mathematically expressed as follows:

(1) $$\begin{array}{c} \begin{array}{c} q\left(y_{t}|y_{t-1}\right)=N\left(y_{t};\sqrt{1-\beta_{t}}y_{t-1},\beta_{t}I\right), \end{array} \end{array}$$

where $y_{t-1}$ represents the image at time step t−1, $y_{t}$ is the noisy image at time step *t*, $\beta_{t}\in \left(0,1\right)$ is a variance, and I is the identity matrix. Given the initial image $y_{0}$, the distribution of $y_{t}$ can be expressed as follows:

(2) $$\begin{array}{c} \begin{array}{c} q\left(y_{t}|y_{0}\right)=N\left(y_{t};\sqrt{\gamma_{t}}y_{0},\left(1-\gamma_{t}\right)I\right), \end{array} \end{array}$$

where $\gamma_{t}=\prod_{i=1}^{t}\left(1-\beta_{i}\right)$. The process proceeds iteratively, adding noise at each time step until the image distribution converges to a standard Gaussian distribution after *T* steps.

In the reverse process, we use the degraded underwater image *x* to estimate the conditional distributions $p_{\theta }\left(y_{t-1}|y_{t},x\right)$. The inference process can be modeled as a reverse Markovian process, aiming to recover the target image $y_{0}$ from the Gaussian noise $y_{T}∼N\left(0,I\right)$, i.e.,

(3) $$\begin{array}{c} \begin{array}{c} p_{\theta }\left(y_{t-1}|y_{t},x\right)=N\left(y_{t-1};\mu_{\theta }\left(y_{t},x,t\right),\sigma_{t}^{2}I\right). \end{array} \end{array}$$

Among them, $p_{\theta }\left(y_{t-1}|y_{t},x\right)$ can be denoted as the combination of $y_{t}$ and a predicted noise $\epsilon_{\theta }\left(y_{t},x,t\right)$ by a neural network. We adopt a U-Net architecture integrated with a multi-scale dynamic-windowed attention module as the denoising network.

#### 3.1.1. Multi-Scale Dynamic-Windowed Attention Module

**Figure 2:**
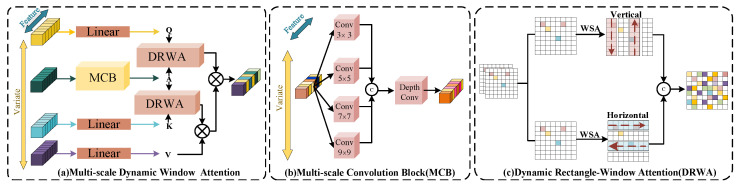
The diagram of the multi-scale dynamic-windowed attention module. (**a**) Multi-scale dynamic-windowed attention, (**b**) multi-scale convolution block (MCB), (**c**) dynamic rectangle-windowed attention (DRWA).

Underwater images often exhibit distinct challenges due to complex environmental conditions, such as water turbidity, light absorption, and scattering effects. These factors create unique visual properties, such as cross-scale similarities (where similar features appear at multiple scales) and anisotropy (directional variation in features, such as water texture or object edges). These characteristics demand specific considerations when extracting features for tasks like underwater image enhancement. In this part, we introduce a multi-scale dynamic-windowed attention module to address these challenges, as illustrated in Figure 2.

To effectively capture cross-scale similarities, we employ the multi-scale Convolution block to extract local features. Specifically, the input feature $X\in R^{C\times H\times W}$ is partitioned into four parts, each maintaining $\frac{C}{4}$ channels, resulting in partitioned feature maps $X_{i}\in R^{\frac{C}{4}\times H\times W}$. Subsequently, convolution kernels of varying sizes (Conv3×3,Conv5×5,Conv7×7,Conv9×9) are applied to the partitioned feature maps to obtain a series of feature maps $X_{i}^{\prime }\in R^{\frac{C}{4}\times H\times W}$ rich in spatial information. This design not only introduces local inductive bias but also comprehensively captures spatial information at different scales. These feature maps are then concatenated along the channel dimension to obtain an agent feature token rich in multi-scale information:

(4) $$\begin{array}{c} \begin{array}{c} A=Cat(\left[X_{1}^{\prime },X_{2}^{\prime },X_{3}^{\prime },X_{4}^{\prime }\right]), \end{array} \end{array}$$

where $A\in R^{C\times H\times W}$. This token not only preserves the local details but also integrates global contextual information across different scales, thereby providing a rich feature representation for subsequent processing.

To effectively address anisotropy caused by factors such as light and water flow, we introduce a dynamic rectangular window attention mechanism. This mechanism divides the input features into a series of non-overlapping rectangular windows $X_{i}\in R^{\frac{C}{4}\times sh\times sw}$, each with a height of sh and a width of sw, where *i* denotes the index of the *i*-th window, resulting in a total of $\frac{H\times W}{sh\times sw}$ windows. Subsequently, we split the feature maps into two groups along the channel dimension and apply attention mechanisms with windows of different directions to each group, which allows us to establish window-based dependencies over a larger range within specific dimensions. Afterward, the two groups of features are reconnected along the channel dimension and interact with the value tokens to aggregate key information.

Specifically, the feature token *A* serves as an agent for the query token *Q*, indirectly interacting with the key (*K*) and value (*V*) tokens to aggregate key information. Finally, the processed information is passed back to the query token *Q* to further enrich its information content. This indirect interaction mode not only substantially reduces the computational burden but also enhances the efficiency of information integration. The interaction process is realized through the following formula:

(5) $$\begin{array}{c} \begin{array}{c} \tilde{S}=Softmax\left(\frac{Q\cdot A^{T}}{\sqrt{d}}\right)\cdot Softmax\left(\frac{A\cdot K^{T}}{\sqrt{d}}\right)\cdot V. \end{array} \end{array}$$

#### 3.1.2. Objective

To enhance local-scale detail recovery and stabilize adversarial training, we adopt a patch-based discriminator *D*. During training only, PatchGAN partitions each generated image into small patches (e.g., 70×70 pixels) and classifies each as real or fake. The adversarial loss function is given as follows:

(6) $$\begin{array}{c} \begin{array}{c} L_{adv}\left(G,D\right)=\arg\min_{G}\max_{D}E_{y_{0}∼p_{data}\left(y_{0}\right)}\left[\log D(y_{0})\right]+\\ E_{x∼p_{data}\left(x\right)}\left[\log(1-D(G(x)))\right]. \end{array} \end{array}$$

The corresponding optimization objective for the diffusion model can be expressed as follows:

(7) $$\begin{array}{c} \begin{array}{c} L_{dif}=\min_{\theta }E_{y_{0}∼q\left(y_{0}\right),x,\epsilon ∼N\left(0,I\right),t}{∥\epsilon -\epsilon_{\theta }\left(y_{t},x,t\right)∥}^{2}. \end{array} \end{array}$$

The comprehensive goal of the proposed model can be expressed as follows:

(8) $$\begin{array}{c} \begin{array}{c} L=L_{adv}\left(G,D\right)+L_{dif}. \end{array} \end{array}$$

We propose an approach that compels the network to preserve realism in every region, ensuring the effective removal of localized underwater haze or chromatic aberrations without sacrificing edge sharpness. Compared with the full-image discriminator, the network structure of PatchGAN is lighter and less computationally intensive, which helps to maintain training stability and suppress excessive smoothing and pattern collapse during the gradual denoising of the diffusion model. Algorithm 1 gives the complete training and inference process for ALDiff-UIE.
**Algorithm 1** ALDiff-UIE Training and Inference**Require:** Paired dataset $\left\{\left(x,y_{0}\right)\right\}$, noise schedule ${\left\{\beta_{t}\right\}}_{t=1}^{T}$**Ensure:** Trained generator $G_{\theta }$ and discriminator $D_{\phi }$
  1: **Training:**  2: **for** each minibatch $\left(x,y_{0}\right)$ **do**  3:      Sample timestep t∼Uniform{1,…,T}  4:      Sample noise ϵ∼N(0,I)  5:      Compute noisy image $y_{t}=\sqrt{\gamma_{t}}\;y_{0}+\sqrt{1-\gamma_{t}}\;\epsilon$  6:      $\hat{\epsilon }=G_{\theta }\left(y_{t},\;x,\;t\right)$  7:      $L_{\mathrm{dif}}={∥\epsilon -\hat{\epsilon }∥}^{2}$  8:      Generate enhancement ${\tilde{y}}_{0}=G_{\theta }\left(x\right)$  9:      $L_{\mathrm{adv}}=-\log D_{\phi }\left(y_{0}\right)\;-\;\log\left(1-D_{\phi }\left({\tilde{y}}_{0}\right)\right)$10:      $\phi \leftarrow \phi +\nabla_{\phi }\;\left(L_{\mathrm{adv}}\right)$             ▹ Update discriminator11:      $\theta \leftarrow \theta -\nabla_{\theta }\;\left(L_{\mathrm{dif}}+L_{\mathrm{adv}}\right)$              ▹ Update generator12: **end for**13: **Inference:**14: Initialize $y_{T}∼N\left(0,I\right)$15: **for** t=T,…,1 **do**16:      $y_{t-1}=\mu_{\theta }\left(y_{t},x,t\right)+\sigma_{t}\;\epsilon_{\theta }\left(y_{t},x,t\right)$17: **end for**18: **return** Enhanced image $y_{0}$


## 4. Experiments

To verify the effectiveness of the proposed method, comprehensive experiments were conducted in this section. Specifically, both qualitative and quantitative experiments were performed, comparing our method with eight state-of-the-art methods. Qualitative evaluation refers to judging the naturalness and visual details of the enhancement effect through visual comparisons (e.g., subjective scoring or side view comparisons). Quantitative evaluation relies on objective metrics (e.g., UICQE [34], PCQI [35], entropy [36], etc.) to measure the degree of improvement in image quality. Ablation studies were conducted to gain insights into the contribution of each component. The following sections provide detailed implementation information and a thorough analysis of the experimental results.

### 4.1. Implementation Details

The proposed model was trained using the underwater image enhancement benchmark (UIEB) dataset [37], which comprises 890 pairs of clear and degraded underwater images. To ensure the effectiveness of the model’s training, we selected 800 pairs of images for training, with the remaining 90 pairs reserved for testing. The training was conducted on an NVIDIA™ GeForce RTX 3090 platform, using the PyTorch [38] framework. Model parameter optimization was carried out with the Adam optimizer, a learning rate of $5\times 10^{-5}$, and a batch size of four for each training iteration. The entire training process consisted of 300 iterations.

To thoroughly assess the generalization capabilities of the proposed model, we conducted a comparative analysis between the proposed method and eight different approaches, as follows: Shallow [39], MLFCGAN [19], MetaUE [40], FUnIE-GAN [20], UIESS [41], DM [31], LiteEnhanceNet [42], and FiveA [16]. This analysis was performed across four datasets, namely, U45 [43], SUIM [44], UIEB [37], and LSUI [25], ensuring a comprehensive and objective evaluation. U45 [43], specifically designed by Li et al. for testing, contains images with color casts, low contrast, and a fog-like effect simulating underwater degradation, providing a realistic representation of underwater environments. SUIM [44] includes a wide variety of underwater images across eight categories, such as fish, coral reefs, plants, sunken ships/ruins, humans, robots, seabed/sand, and water backgrounds. LSUI [25] serves as a large-scale underwater image repository, comprising 4279 underwater images along with corresponding high-quality reference images, offering ample validation resources for assessing the model performance.

For quantitative evaluation, we use widely recognized objective quality assessment metrics to assess images, which provide precise evaluation functions for aspects such as color, detail, and contrast. UIQM [45], a comprehensive evaluation metric, is a linear combination of three metrics, namely, UICM, USIM, and UIConM. A higher UIQM value indicates better image quality. UCIQE [34] assesses images in the CIELAB color space, with a higher UCIQE value signifying a better balance in chromaticity, contrast, and saturation enhancement. PCQI [35] quantifies image distortion within individual blocks by evaluating the average intensity, signal strength, and signal structure. A higher PCQI value indicates better image quality. The gradient-based contrast evaluation index contrast_r [46] reflects the contrast of the image, with higher values indicating greater contrast. Entropy [36] quantifies the information content of an image, with higher values indicating greater information richness.

In ablation experiments, since the UIEB dataset provides reference images, we utilize full-reference image quality assessment metrics, specifically the peak signal-to-noise ratio (PSNR) [47] and the structural similarity index (SSIM) [48], to quantify the performance of each component. PSNR measures the difference between the enhanced image and the reference image, with a higher value indicating a smaller difference and better image quality. SSIM evaluates the similarity between the enhanced image and the reference image, with a higher SSIM value indicating higher structural similarity. Additionally, we utilize CIEDE2000 [49] for color quality evaluation, considering factors such as the human eye’s sensitivity to different color stimuli and the scope of color difference calculations. CIEDE2000 provides a more accurate color quality assessment, with lower values indicating higher color quality.

### 4.2. Qualitative Comparisons

**Figure 3:**
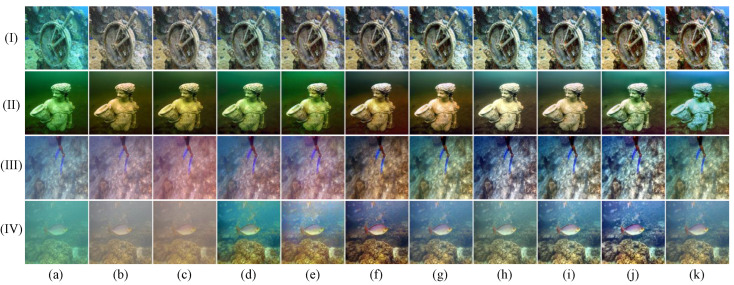
Qualitative comparisons for samples from the UIEB dataset: I-IV represents the four images in the UIEB dataset. From left to right are (**a**) raw underwater images, the results of (**b**) shallow [39], (**c**) MLFCGAN [19], (**d**) MetaUE [40], (**e**) FUnIE [20], (**f**) UIESS [41], (**g**) DM [31], (**h**) LiteEnhanceNet [42], (**i**) FiveA [16], and (**j**) the proposed method, and (**k**) reference images.

**Figure 4:**
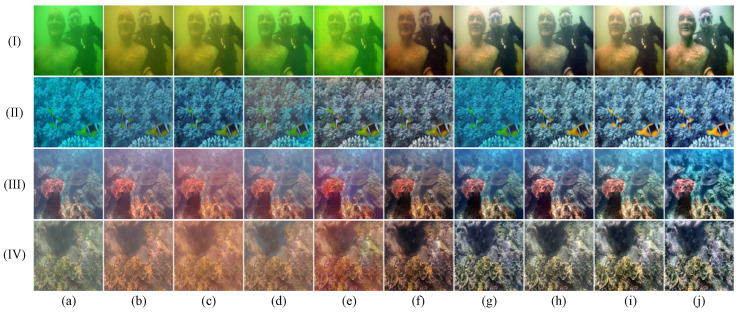
Qualitative comparisons for samples from the U45 dataset: I–IV represents the four images in the U45 dataset. From left to right are (**a**) raw underwater images, the results of (**b**) shallow [39], (**c**) MLFCGAN [19], (**d**) MetaUE [40], (**e**) FUnIE [20], (**f**) UIESS [41], (**g**) DM [31], (**h**) LiteEnhanceNet [42], (**i**) FiveA [16], and (**j**) the proposed method.

**Figure 5:**
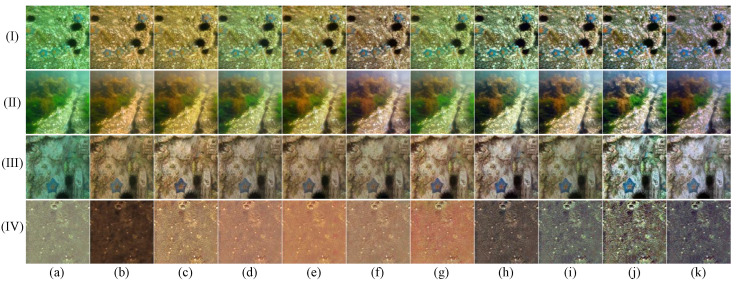
Qualitative comparisons for samples from the LSUI dataset: I–IV represents the four images in the LSUI dataset. From left to right are (**a**) raw underwater images, the results of (**b**) shallow [39], (**c**) MLFCGAN [19], (**d**) MetaUE [40], (**e**) FUnIE [20], (**f**) UIESS [41], (**g**) DM [31], (**h**) LiteEnhanceNet [42], (**i**) FiveA [16], (**j**) the proposed method, and (**k**) reference images.

**Figure 6:**
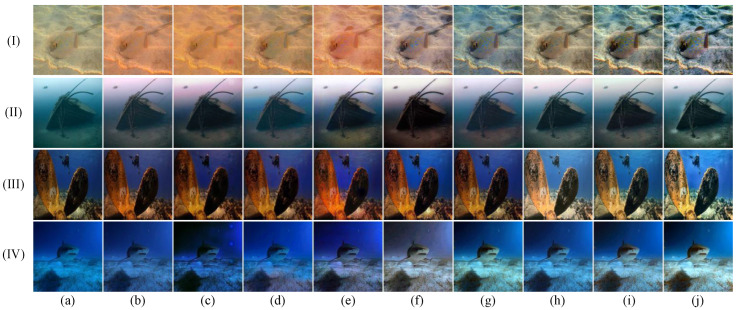
Qualitative comparisons for samples from the SUIM dataset: I–IV represents the four images in the SUIM dataset. From left to right are (**a**) raw underwater images, the results of (**b**) shallow [39], (**c**) MLFCGAN [19], (**d**) MetaUE [40], (**e**) FUnIE [20], (**f**) UIESS [41], (**g**) DM [31], (**h**) LiteEnhanceNet [42], (**i**) FiveA [16], and (**j**) the proposed method.

In this section, we conduct a thorough qualitative comparison utilizing publicly available underwater image datasets to evaluate the generalization performance of the proposed method in real-world scenarios. The comparative results, shown in Figure 3, Figure 4, Figure 5 and Figure 6, clearly highlight the distinct performances of various methods across the UIEB, U45, LSUI, and SUIM datasets.

As shown in Figure 3, in cases (I), (III), and (IV), our method produces more realistic colors and richer details compared to other approaches. In case (II), the FiveA method shows a certain advantage in color fidelity, yet our method preserves a greater amount of image details.

As depicted in Figure 4, in scenes (I) and (II), our method preserves rich color information and effectively maintains image details, while the other methods exhibit varying degrees of color deviation. In scene (III), although the LiteEnhanceNet method achieves more natural color reproduction, our method demonstrates a significant advantage in detail recovery. In scene (IV), both the DM method and our method achieve accurate color restoration in underwater environments, but our method renders finer, sharper details.

As can be seen from Figure 5, in scene (I), while both LiteEnhanceNet and our method render natural colors, our method demonstrates a notable advantage in contrast enhancement and detail presentation. In scenes (II), (III), and (IV), our method not only accurately restores the images’ colors but also delivers superior clarity and contrast.

As illustrated in Figure 6, scenes (I), (II), and (IV) demonstrate the superior ability of the proposed method to accurately restore colors, effectively mitigating color deviations compared to other methods. However, in scene (III), although LiteEnhanceNet, FiveA, and our method all produce natural colors, a closer inspection reveals that LiteEnhanceNet is overexposed, while FiveA falls short of our method in terms of detail precision.

### 4.3. Quantitative Comparisons

In this section, we conduct a comprehensive quantitative comparison between the proposed method and several state-of-the-art image enhancement methods. Table 1 provides evaluation results for each enhanced image shown in Figure 3 and Figure 4. It is evident that our method outperforms others across three evaluation metrics, namely, UCIQE, PCQI, and entropy. Notably, in scenario (II) of Figure 3, DM achieved the highest UCIQE score, yet its enhanced images exhibited a significant red color cast. In scenario (II) of Figure 3 and scenario (IV) of Figure 4, FiveA achieved higher entropy scores; however, the details of its enhanced results were relatively blurred.

Table 2 showcases the quantitative evaluation results for the enhanced images in Figure 5 and Figure 6, further validating the comprehensive superiority of our method. While other methods occasionally outperformed ours in specific metrics for certain scenarios, these improvements were often accompanied by issues like color casts. For instance, in scenario (II) of Figure 6, FUnIE and MLFCGAN obtain the best UCIQE scores, yet the enhanced images of both methods suffer from a red color bias. In scenario (IV), although FiveA achieves a higher entropy score, its resulting image exhibits an undeniable blue bias. It must be emphasized that while existing no-reference image quality assessment metrics have provided essential benchmarks for evaluating image quality, studies [50,51] have demonstrated that these metrics may exhibit biases towards certain specific image characteristics. Consequently, relying solely on quantitative evaluations is insufficient for a comprehensive and accurate assessment of model quality. It is imperative to integrate both qualitative and quantitative analyses for a more holistic evaluation.

**Figure 7:**
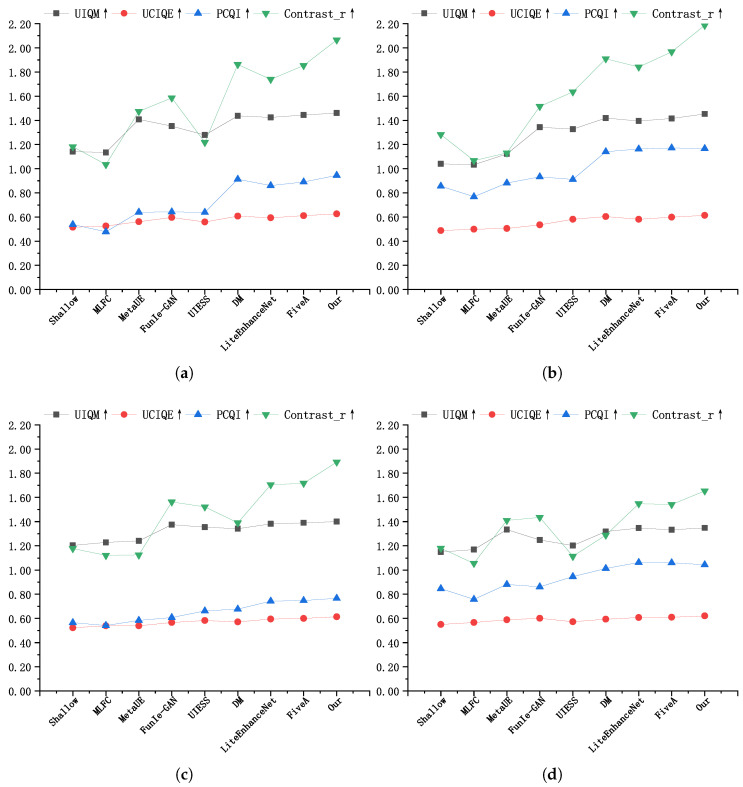
Average quantitative values on different datasets. (**a**) Average quantitative values on the UIEB dataset. (**b**) Average quantitative values on the U45 dataset. (**c**) Average quantitative values on the LSUI dataset. (**d**) Average quantitative values on the SUIM dataset.↑ indicates that higher is better.

Additionally, Figure 7 presents the average values of UIQM, UCIQE, PCQI, and other metrics across the UIEB, U45, LSUI, and SUIM datasets, respectively. From these figures, it can be clearly observed that the proposed method outperforms other methods across all evaluated metrics. These results demonstrate that the proposed method efficiently enhances images across various underwater environments, validating its effectiveness and generalizability.

To evaluate the complexity of our proposed method, we conducted experiments on the UIEB dataset, following the experimental conditions outlined in the implementation details. As shown in Table 3, our method does not outperform existing approaches in terms of model size or latency. This is primarily because we incorporate a multi-stage reverse-diffusion module and discriminator with inter-stage feature interactions and attention weighting, which balances global structure restoration and local detail refinement. We also employ multiple reconstruction and adversarial losses and perform additional intermediate-feature fusion and enhancement during inference to better restore texture and color. Although these designs improve image quality, they introduce numerous convolutional layers and attention operations, which increase both the parameter count and inference time.

### 4.4. Ablation Study

In this section, we design an ablation experiment to verify the effectiveness of the individual components within the proposed model. We use Palette [52] as the baseline model for our experiments, and the specific ablation experiments are as follows:

- woMDWA: Removing the multi-scale dynamic-windowed attention mechanism from the proposed model to investigate its key role in the image enhancement task.
- woDIS: Removing the discriminator from the proposed model to evaluate its impact on the overall model performance.

**Figure 8:**
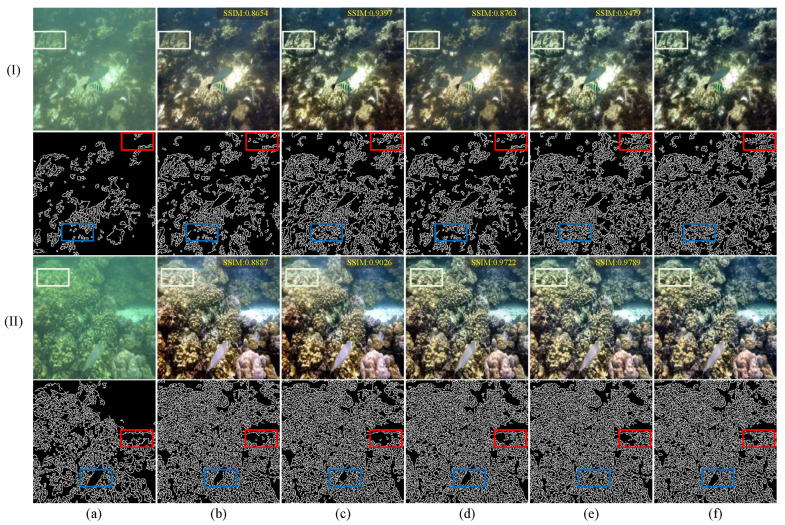
Visual comparison of the ablation study sampled from the UIEB dataset. (I) and (II) represent two images from the UIEB dataset. White, red, and blue rectangles are used to mark key areas in the image. From left to right are (**a**) raw underwater images, the results of (**b**) Baseline [52], (**c**) woMDWA, (**d**) woDIS, and (**e**) the proposed method, and (**f**) reference images.

**Figure 9:**
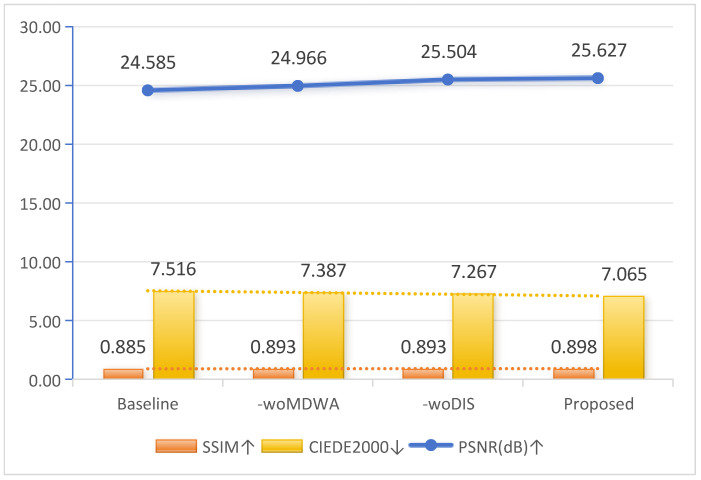
Ablation study of different components on the UIEB dataset. ↑ indicates higher is better, ↓ indicates lower is better.

Figure 8 presents the results of the ablation study intuitively, demonstrating the impact of various components on the final enhancement outcome. White boxes mark identical key regions in the color images, facilitating side-by-side comparison of color restoration and contrast. Red boxes enclose the corresponding areas in the Canny edge maps, highlighting how each variant preserves primary structural edges (e.g., coral ridges, fish contours). Blue boxes indicate areas of fine-grained texture. Comparative analysis suggests that the discriminator ensures a balanced overall color distribution, while the multi-scale dynamic-windowed attention mechanism directs focus to target regions. The Canny-based edge maps further confirm that our complete method not only retains the main edges (red boxes) but also uncovers richer peripheral textures (blue boxes). Moreover, Figure 9 displays the average evaluation results of different components on the UIEB dataset. The proposed method achieved the best performance across all evaluation metrics, demonstrating its effectiveness.

## 5. Discussion

ALDiff-UIE introduces, for the first time, a combination of progressive detail reconstruction via diffusion models and adversarial learning, yielding significant gains in detail-recovery metrics such as PCQI while maintaining color consistency. Its multi-scale dynamic-windowed attention module surpasses fixed-window and single-scale attention mechanisms in capturing anisotropic and cross-scale textures.

Despite its leading performance in underwater image enhancement, ALDiff-UIE exhibits three primary limitations. First, its generalization to unseen water types (e.g., highly turbid river water or deep-sea environments) remains limited; future work may incorporate domain-adaptive or unsupervised fine-tuning strategies to improve adaptability across diverse water conditions. Second, unreferenced metrics such as UCIQE and PCQI may not align with subjective human perception in all scenarios, so adopting more robust perceptual-quality indicators or conducting large-scale subjective evaluations could yield more reliable assessments. Third, the combination of multi-stage backpropagation, multi-scale dynamic-windowed attention, and a PatchGAN discriminator substantially increases parameter count and inference cost, hindering real-time deployment in resource-constrained settings. Thus, future research will explore lightweight architectures, dynamic inference strategies, and more efficient unsupervised adaptation methods to enhance the model’s utility and robustness.

## 6. Conclusions

In this paper, we propose ALDiff-UIE, an underwater image enhancement method that integrates adversarial learning with diffusion modeling and incorporates a multi-scale dynamic-windowed attention mechanism to effectively capture cross-scale similarity and anisotropy in underwater images. Qualitative and quantitative experiments on four benchmark datasets—UIEB, U45, SUIM, and LSUI—demonstrate that ALDiff-UIE increases the average PCQI by approximately 12.8% and UIQM by about 15.6%. It also significantly outperforms existing methods on several no-reference metrics, including UCIQE and entropy (*p* < 0.01), and visual comparisons reveal more natural color reproduction and richer detail. Despite these improvements, ALDiff-UIE remains computationally intensive, with notable inference latency, and its performance degrades in extremely turbid or low-light conditions. In the future, we will compress the model and accelerate the inference through structured pruning, low-bit quantization, and knowledge distillation, and introduce unsupervised or semi-supervised domain adaptive fine-tuning strategies to maintain excellent generalization ability in various water environments such as high turbidity rivers and deep-sea low light, to satisfy the demand for real-time deployment on embedded and mobile devices.

## Figures and Tables

**Table 1:** Quantitative evaluation results of different methods on the sample images presented in Figure 3 and Figure 4; ↑ indicates that higher is better.

Metrics	Methods	Figure 3				Figure 4			
		(I)	(II)	(III)	(IV)	(I)	(II)	(III)	(IV)
UCIQE ↑	Shallow [39]	0.535	0.616	0.448	0.391	0.535	0.458	0.497	0.448
	MLFCGAN [19]	0.550	0.628	0.455	0.382	0.550	0.504	0.483	0.407
	MetaUE [40]	0.565	0.637	0.519	0.555	0.565	0.503	0.526	0.452
	FUnIE [20]	0.579	0.613	0.494	0.548	0.579	0.519	0.561	0.485
	UIESS [41]	0.592	0.652	0.585	0.615	0.592	0.514	0.589	0.560
	DM [31]	0.626	**0.658**	0.570	0.530	0.626	0.475	0.621	0.561
	LiteEnhanceNet [42]	0.615	0.646	0.585	0.504	0.615	0.542	0.629	0.545
	FiveA [16]	0.622	0.639	0.604	0.586	0.622	0.560	0.632	0.603
	Proposed	**0.649**	0.638	**0.607**	**0.621**	**0.649**	**0.579**	**0.639**	**0.592**
PCQI ↑	Shallow [39]	0.602	0.504	0.556	0.529	0.602	0.987	0.797	0.744
	MLFCGAN [19]	0.539	0.433	0.467	0.489	0.539	0.972	0.665	0.581
	MetaUE [40]	0.729	0.587	0.631	0.888	0.729	1.020	0.834	0.827
	FUnIE [20]	0.667	0.737	0.718	0.344	0.732	0.945	0.771	0.999
	UIESS [41]	0.650	0.453	0.676	0.924	0.650	1.165	0.902	0.821
	DM [31]	0.886	0.717	0.960	0.970	0.886	1.094	1.111	1.306
	LiteEnhanceNet [42]	0.948	0.817	1.031	0.997	0.948	1.246	1.222	1.246
	FiveA [16]	0.959	0.766	1.098	1.137	0.959	1.235	1.250	1.302
	Proposed	**0.976**	**0.936**	**1.237**	**1.283**	**0.976**	**1.276**	**1.324**	**1.343**
Entropy ↑	Shallow [39]	7.490	7.139	6.831	6.147	7.490	7.406	7.051	7.030
	MLFCGAN [19]	7.579	7.147	6.997	6.443	7.579	7.496	7.192	7.255
	MetaUE [40]	7.607	7.391	7.048	7.306	7.607	7.233	7.051	6.999
	FUnIE [20]	7.689	7.301	7.107	7.312	7.689	7.403	7.357	7.392
	UIESS [41]	7.667	7.225	7.400	7.346	7.667	7.429	7.413	7.370
	DM [31]	7.847	7.479	7.484	7.123	7.847	7.541	7.673	7.533
	LiteEnhanceNet [42]	7.849	7.525	7.694	7.008	7.849	7.694	7.685	7.490
	FiveA [16]	7.876	**7.589**	7.750	7.461	7.876	7.767	7.733	**7.774**
	Proposed	**7.889**	7.530	**7.751**	**7.658**	**7.889**	**7.859**	**7.876**	7.772

**Table 2:** Quantitative evaluation results of different methods on the sample images presented in Figure 5 and Figure 6, ↑ indicates higher is better.

Metrics	Methods	Figure 5				Figure 6			
		(I)	(II)	(III)	(IV)	(I)	(II)	(III)	(IV)
UCIQE ↑	Shallow [39]	0.487	0.544	0.556	0.357	0.399	0.544	0.618	0.471
	MLFCGAN [19]	0.533	0.568	0.564	0.379	0.402	0.590	0.651	0.500
	MetaUE [40]	0.480	0.541	0.557	0.358	0.414	0.564	0.623	0.489
	FUnIE [20]	0.533	0.568	0.564	0.379	0.402	**0.590**	0.651	0.500
	UIESS [41]	0.544	0.579	0.583	0.421	0.478	0.587	0.656	0.539
	DM [31]	0.492	0.551	0.552	0.447	0.496	0.566	0.632	0.581
	LiteEnhanceNet [42]	0.559	0.599	0.620	0.452	0.487	0.577	0.661	0.534
	FiveA [16]	0.576	0.601	0.630	0.537	0.578	0.577	0.671	0.554
	Proposed	**0.582**	**0.616**	**0.635**	**0.558**	**0.583**	0.578	**0.692**	**0.617**
PCQI ↑	Shallow [39]	0.550	0.693	0.678	0.252	0.632	0.940	0.744	0.953
	MLFCGAN [19]	0.509	0.626	0.660	0.188	0.534	0.833	0.584	0.827
	MetaUE [40]	0.594	0.797	0.749	0.359	0.917	0.978	0.879	0.999
	FUnIE [20]	0.667	0.737	0.718	0.344	0.732	0.945	0.771	0.999
	UIESS [41]	0.757	0.828	0.759	0.473	0.981	0.903	0.787	0.984
	DM [31]	0.611	0.890	0.815	0.661	1.151	0.973	1.014	1.047
	LiteEnhanceNet [42]	0.922	1.108	0.949	0.694	1.219	1.069	0.947	1.072
	FiveA [16]	0.941	1.070	0.933	0.882	1.322	1.068	1.029	1.061
	Proposed	**1.079**	**1.140**	**1.097**	**0.955**	**1.390**	**1.111**	**1.076**	**1.033**
Entropy ↑	Shallow [39]	7.183	7.674	7.747	6.828	7.364	7.539	7.090	7.459
	MLFCGAN [19]	7.314	7.692	7.772	6.737	7.302	7.491	6.979	7.033
	MetaUE [40]	7.197	7.540	7.652	6.850	7.295	7.555	7.114	7.285
	FUnIE [20]	7.543	7.686	7.794	7.119	7.467	7.615	7.308	7.474
	UIESS [41]	7.532	7.629	7.732	6.331	7.062	7.446	7.338	7.522
	DM [31]	7.168	7.606	7.665	6.473	7.009	7.513	7.191	7.643
	LiteEnhanceNet [42]	7.666	7.787	7.883	6.876	7.154	7.663	7.715	7.525
	FiveA [16]	7.723	7.771	7.864	7.276	7.514	7.597	7.541	**7.686**
	Proposed	**7.846**	**7.826**	**7.907**	**7.418**	**7.633**	**7.799**	**7.853**	7.602

**Table 3:** Comparison of the number of parameters and the single image testing time of each method.

Methods	Params (M)	Testing per Image (s)
Shallow [39]	7.40 M	0.05
MLFCGAN [19]	565.6 M	0.06
MetaUE [40]	24.42 M	0.49
FUnIE [20]	7.02 M	0.135
UIESS [41]	4.26 M	0.006
DM [31]	10 M	0.16
LiteEnhanceNet [42]	1.64 M	0.114
FiveA [16]	0.01 M	0.03
Proposed	65.59 M	22

## Data Availability

Data underlying the results presented in this paper are available in the UIEB dataset [37], U45 dataset [43], SUIM dataset [44], and LSUI dataset [25].
